# Amplitude distribution of stochastic oscillations in biochemical networks due to intrinsic noise

**DOI:** 10.1186/1757-5036-2-10

**Published:** 2009-11-17

**Authors:** Moritz Lang, Steffen Waldherr, Frank Allgöwer

**Affiliations:** 1 Institute for Systems Theory and Automatic Control, Universität Stuttgart, Pfaffenwaldring 9, 70569 Stuttgart, Germany

## Abstract

Intrinsic noise is a common phenomenon in biochemical reaction networks and may affect the occurence and amplitude of sustained oscillations in the states of the network. To evaluate properties of such oscillations in the time domain, it is usually required to conduct long-term stochastic simulations, using for example the Gillespie algorithm. In this paper, we present a new method to compute the amplitude distribution of the oscillations without the need for long-term stochastic simulations. By the derivation of the method, we also gain insight into the structural features underlying the stochastic oscillations. The method is applicable to a wide class of non-linear stochastic differential equations that exhibit stochastic oscillations. The application is exemplified for the MAPK cascade, a fundamental element of several biochemical signalling pathways. This example shows that the proposed method can accurately predict the amplitude distribution for the stochastic oscillations even when using further computational approximations.

**PACS Codes: **87.10.Mn, 87.18.Tt, 87.18.Vf

**MSC Codes: **92B05, 60G10, 65C30

## 1. Introduction

Oscillations are a widely occurring phenomenon in the dynamics of biological systems. On the intracellular level, oscillations occur for example in the activity of various genes or signalling proteins. To gain more insight into the processes related to these oscillations, mathematical models for the underlying biochemical and genetic networks are commonly constructed. Such models have proven helpful in connecting the underlying biochemistry with the temporal characteristics of emerging oscillations and the associated biological function. Examples for this type of results include the intracellular circadian clock [1] or the developmental process of somitogenesis [2].

In dynamical models for biological systems, oscillations are due to limit cycles or more complex attractors in deterministic frameworks, or may emerge from various stochastic effects in stochastic frameworks. Concerning the biological function, one would not expect that it makes a significant difference whether oscillations are due to a deterministic limit cycle or arise from stochastic effects. For the biological function, rather the temporal characteristics of oscillations are relevant, such as their frequency or amplitude. For deterministic limit cycle oscillations, these characteristics are easily computed by a numerical simulation of the model. For stochastic models, computing the temporal characteristics of oscillations is much more involved. Two approaches are common for solving stochastic systems. In the first approach, the so called chemical master equation (CME) is used [3]. The solution to the CME (or approximations thereof) yields the probabilities for each of the possible configurations of the system to be attained at a given point in time. However, since the temporal correlations of these probabilities are usually not obtained from the CME, the temporal characteristics of oscillations are not directly accessible from such a solution. In the second approach, a large number of realisations of the stochastic process are computed. These realisations can then be used to compute various temporal characteristics of the system, in particular oscillation amplitude and frequency distributions. Yet, the computation of a sufficiently large number of realisations typically entails a large computational effort.

In this paper, we develop a new method to compute the amplitude distribution for systems exhibiting stochastic oscillations. We thereby focus on systems where the deterministic part has a weakly stable equilibrium point (EP), typically with damped oscillations, and the stochastic effects induce sustained oscillations around this EP. In such systems, we can distinguish two mechanisms by which stochastic oscillations occur. For the first mechanism, the system needs to have the property that a certain small perturbation away from the equilibrium point leads to a large excursion in the state space before the system returns to the proximity of the EP [4]. If the noise is strong enough to reliably generate such a perturbation, but does not disturb the system during the round trip too much, we recognize regular oscillations with a well defined amplitude and frequency. This effect is called Coherence Resonance [5] or Stochastic Resonance [6, p. 149 ff.]. For the second mechanism, the deterministic part of the system already gives rise to damped oscillations, and the noise just serves to sustain the oscillations [7,8]. In this case, the density distribution of realizations in the state space will typically be similar to a Gaussian-like distribution with a maximum at the EP, while individual trajectories show pronounced oscillations with a frequency close to the frequency of the deterministic part. The amplitudes of this type of oscillations increase with the noise power, in contrast to the other type of systems, which mainly exhibit oscillations of a fixed amplitude independent of the noise power.

For the second mechanism, it is of interest to compute the amplitude distribution of the stochastic oscillations. Several methods to solve this problems were developed previously. In [9] the average change of a stochastic Lyapunov function was determined. By setting this average change to zero one may identify an orbit around which a stochastic realization fluctuates, and thereby estimate a typical amplitude. In the context of this article the results of Kuske et al. [7,8] are very interesting. They apply a multi-scale ansatz to separate the properties of the oscillations mainly determined by the deterministic part of the analyzed system (the frequency) from those determined to a high percentage by the stochastic part (the amplitude). Therewith it is possible to calculate both the amplitude distribution and the mean frequency of the oscillations. Nevertheless this method has the disadvantage of requiring the discussed systems to be almost linear and the frequency of the oscillations not to be noteworthy disturbed by the stochastic effects.

In this article we focus on biochemical systems that can be modeled as a set of interconnected, possibly nonlinear, stochastic differential equations (SDEs). With the help of the Fokker-Planck equation (see e.g. [3, p. 193 ff.] or [10, p. 120 ff.]), we calculate the stationary density distribution in the state space. We provide a theorem allowing to calculate the amplitude distribution with only the knowledge of the density distribution and the corresponding SDE. The theorem allows not only to consider linear, but also a wide class of non-linear systems and therefore makes it possible to analyze not only systems with oscillations being of low amplitude compared to average concentrations but also of intermediate and high amplitudes. Although stochastic oscillations are a mainly two dimensional effect, we show that they also occur in higher order systems and give an example on how to analyze them then. As far as we are aware of, this paper introduces for the first time a method to analytically analyse higher order possibly non-linear systems being affected with intermediate to high amounts of noise, and thereby showing stochastic oscillations.

The paper is structured as follows. To give an easily accessible introduction to the material, we first present our results on the calculation of the amplitude distribution of stochastic oscillations and afterwards state the underlying assumptions and theorems. Then we explain the application of our theory by calculating the amplitude distribution of a simple example, the damped harmonic oscillator in the presence of additive noise, to get a first insight into the reasons for stochastic oscillations. We want to remark that this example simplifies, due to its structure, the necessary calculations significantly. As a more realistic example, we discuss oscillations in the MAP kinase cascade with an incorporated negative feedback with limited amounts of entities of each molecular species. For this system, the stationary probability distribution can be estimated by a linear approximation, or it can be computed numerically. We compare the amplitude distributions predicted by our method, based on these two approaches, to an "experimental" amplitude distribution obtained from a long-term stochastic simulation.

## 2. Results and Discussion

### 2.1. Amplitude Distribution of the Stochastic Oscillations

In this article we develop a method to derive the amplitude distribution of stochastic oscillations from the knowledge of the stationary density distribution of a stochastic differential equation (SDE). Here we first give a short outline of our results for which we present the corresponding theorems and proofs in the next sections.

Biochemical networks with stochastic dynamics can be modelled by the Langevin equation [11]. For the derivation of the theoretical results, we consider a two-dimensional Langevin equation having an equilibrium point at zero:(1)

with the state vector **x **∈ ℛ^2^, the system's dynamics **f **= (*f*_1_, *f*_2_)^*T*^, where *f*_1 _and *f*_2 _are smooth functions (∈ *C*^∞^), **Σ **a 2-by-2 matrix of smooth functions and **Γ **= (Γ_1_, Γ_2_)^*T*^, where Γ_1 _and Γ_2 _are uncorrelated, statistical independent Gaussian white noise with zero mean and variance of one.

Let us denote the stationary density distribution of the system (1) by *P *(**x**), and the amplitude distribution for the oscillations by *P*_*A*_(χ), where χ denotes the oscillation amplitude. Under certain assumptions (see following sections) the amplitude distribution *P*_*A*_(χ) then satisfies(2)

with *ν*(**x**) = *||***f**(**x**)*|| *the average speed at state **x **and (*χ*) given by(3)

Hereby *A *is defined as *A *= {**x **∈ ℛ^2^|⟨ **x**|⟩ = *χ *} and  is the unit vector in the direction where the amplitude is measured.

The product on the right hand side of (2) represents the steady state flux of reactions through a certain state . Intuitively speaking,  is chosen so that most of the realizations going through  will also reach their maximal value in a small neighborhood of , so that under certain assumptions (see following sections) it is justifiable to approximate the amplitude of such a realization by the value reached at .

The apparent complicated definition of  is a result of the freedom of choice in which direction  the amplitude is measured. In a biological system it is obviously important to distinguish whether one measures the oscillations of the concentration of species *A *or of species *B*. Sometimes it is experimentally not possible to distinguish between two species, e.g. if *B *represents the phosphorylated version of a protein *A*, so that one can only measure the amplitude of [*A*] + [*B*]. Additionally, for nonlinear networks, the amplitude distribution may be different depending on whether one measures the amplitude of the oscillations in the positive direction from the steady state or in the negative direction. If one e.g. measures the amplitude distribution of the concentration of the first species *x*_1 _in the positive direction, (3), i.e.  = (1, 0)^T^, can be simplified to .

To normalize the probability distribution *P*_*A *_of the amplitude, we have to divide equation (2) through the integral of the probability of all amplitudes:(4)

Simple models may have additional properties allowing us to simplify (2). For linear systems the speed *ν*((*χ*)) is directly proportional to *||*(*χ*)*|| *simplifying (2) to(5)

with  given as above.

We want to emphasize here that (2) allows to analytically or numerically calculate the amplitude distribution only from the knowledge of certain properties of the SDE describing the biological system without the necessity to run numerical simulations. The formula yields a good approximation for a wide class of nonlinear problems and can be used to calculate the amplitude distribution even for certain systems having more than two dimensions as we demonstrate in the second example of this paper.

### 2.2. Derivation of the Results

To derive the results presented in the proceeding section we analyze nonlinear SDE systems of order two as given by (1), with an asymptotically stable equilibrium point and damped oscillations in the deterministic part. We restrict our analyis to two dimensional systems, because many higher dimensional systems can be reduced to two dimensions for the purpose of analysing stochastic oscillations. In the derivation we assume w.l.o.g. that we want to compute the amplitude of the oscillations in the positive direction of *x*_1_, i.e.  = (1, 0)^T^, and that the equilibrium point of the deterministic formulation ( = **f**(**x**)) of (1) is at the origin. Both assumptions can easily be satisfied for an arbitrary system by an appropriate coordinate transformation.

We establish an angular phase relationship of the state vector **x**(*t*) with respect to a fixed reference vector in the state space [12]. An oscillation period of the system (1) is defined as the time during which the angle between the state vector and the fixed reference vector changes by 2*π*. Further, the amplitude of an oscillation is defined as the maximal value of *x*_1 _during the corresponding oscillation period.

We make the following assumptions on the system (1):

1. There exists a stationary density distribution *P*: ℛ^2 ^→ ℛ^+^: **x **↦ *P *(**x**) for (1) which is sufficiently smooth in **x**. We demand the curvature of the level curves of this distribution to exist and not to change its sign. For the computation of the amplitude distribution *P*_*A*_(*χ*), *P *(**x**) may be computed analytically, numerically, or may be obtained by the long term limit of a measurement.

2. The deterministic formulation ( = **f**(**x**)) of (1), which is at the state := **x**(*t*_0_) at time *t *= *t*_0 _with the probability of the state  being *P *() =: , will approximatively evolve tangential to the level curve of the probability distribution, thus for small Δ*t *the probability of the state at time *t *+ Δ*t *won't have changed significantly (*P *(**x**(*t*_0 _+ Δ*t*)) ≈ ) This assumption can be easily checked by calculating the Lie derivative of P with respect to **f**:(6)

which has to be small for almost all **x **∈ ^2^ having a non-negligible probability *P *(**x**).

3. In addition we require that the average speed *ν *= *||***f**(**x**)*|| *of (1) does not vanish for almost all **x **∈ ℛ^2 ^having a non-negligible probability *P *(**x**).

Assumption 3 is obviously not satisfied in a small area around the EP where for most systems *ν *becomes very small. However when the system is very close to the EP, the motion of most realizations can be approximated by a random walk. Thus during the time when the system is in the small neighborhood of the EP it is not justifiable to speak about oscillations anymore until the system leaves the neighborhood again. Away from the EP, *||***f**(**x**)*|| *is significantly bigger than zero for most biological relevant systems so that Assumption 3 is fulfilled.

**Figure 1:**
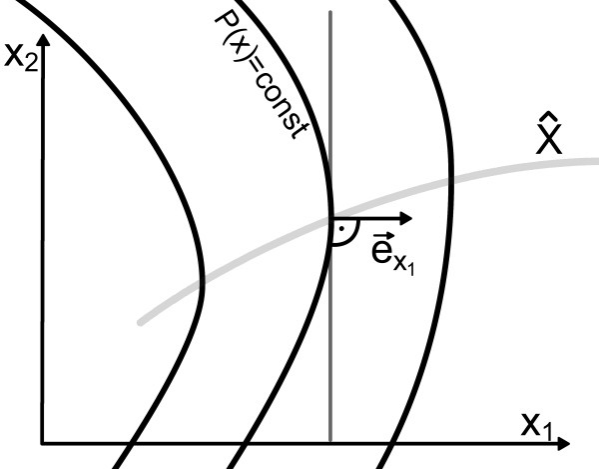
**Definition of **. The solid black lines are the level curves of *P *(**x**). In  ∈  the unit vector  in the direction of *x*_1 _is orthogonal to the tangent on the level curve trough .

With the help of these assumptions we are able to formulate a theorem for the calculation of the amplitude distribution of stochastic oscillations. For the formulation of the theorem let us define(7)

i.e. the unit vector  in the direction of *x*_1 _is orthogonal to the tangent on the level curve of the probability distribution in  (see Figure 1). Because of Assumption 1 there exists exactly one state  ∈  for every amplitude *χ *satisfying  = *χ*. Furthermore we define the following variables, which can be calculated with the knowledge of the system (1) and the stationary probability distribution *P*:(8a)

**Figure 2:**
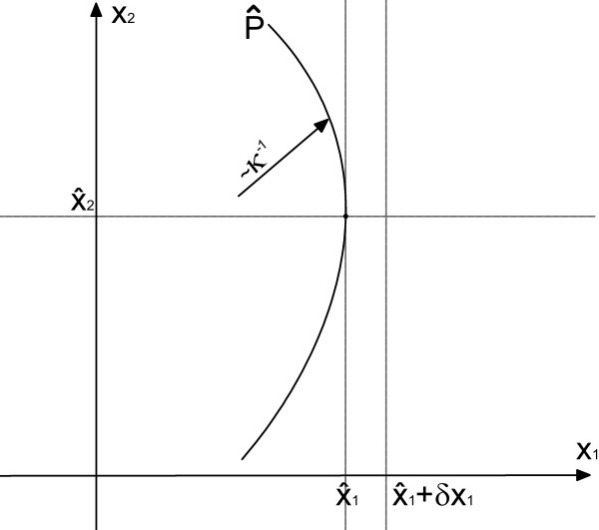
**Visualization of the variables**. Visualization of the variables needed to calculate the amplitude distribution of stochastic oscillations.

The variable *ν *can be thought of as the deterministic speed, *L *is the standard deviation of the Lie-derivative of *P *with respect to the right hand side of (1), *a *the derivative of *P *in the direction of *x*_1_, and *κ *the curvature of *P *with respect to *x*_2_, each evaluated at the states  ∈ . The variables are illustrated in Figure 2.

With the help of these definitions we can formulate the following two lemmas and a theorem, which characterizes the amplitude of the stochastic oscillations.

**Lemma 1 ***Assume the Assumptions 1-3 are satisfied. Let Ψ_*f *_be a realization of (1) being at the state  ∈  at time t_0_. Then the amplitude of Ψ_*f *_during the current oscillation will lie with a probability of 70.8% in the set *[,  + *δx*_1_] *and with a probability of 95.5% in the set [,  + ], with δx_1 _defined by *.

**Lemma 2 ***Assume the Assumptions 1-3 are satisfied. Then the net flux density *(**x**) *of realizations at the state ***x **∈ ℛ^2 ^*has an absolute value proportional to the product of the probability P *(**x**) *and the average speed v*(**x**) *of (1) and is tangential to the level curve of the density distribution P at this point*.

**Figure 3:**
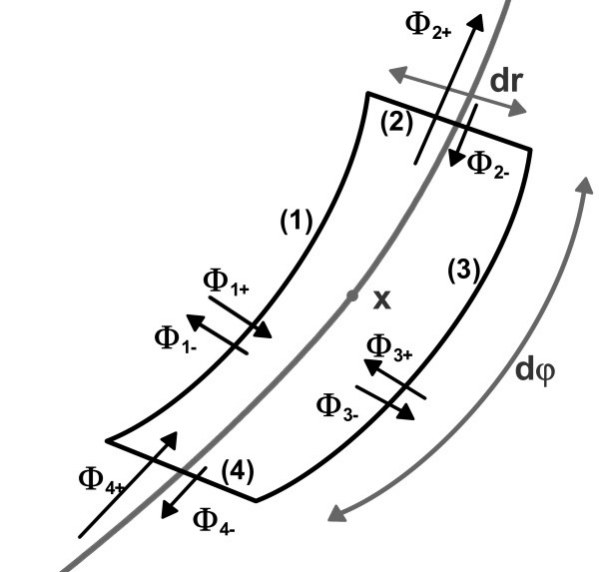
**Flux through unit cell**. The fluxes through the edges of an infinitesimal small unit cell around a state **x **∈ ^2^.

Lemma 2 is derived by considering the flux of realizations through an infinitesimaly small region around the state *x*, as illustrated in Figure 3.

**Theorem 1 (Distribution of amplitudes) ***Under the Assumptions 1-3 and if δx_1_ as defined in Lemma 1 is small, the probability P*_*A*_(*χ*) *of an amplitude χ of the stochastic oscillations of (1) in the direction x*_1 _*is proportional to the absolute value of the net flux density |||| at the state * ∈ *satisfying * = *χ*.

Our results for the calculation of the amplitude distribution *P*_*A*_(*χ*) presented in the previous section (2) directly follow from the application of Theorem 1. The proofs of the respective lemmas and theorem can be found in the appendix.

### 2.3. Remarks

• To check if the assumptions necessary for the application of Theorem 1 are fulfilled, one can calculate *δx*_1 _according to the formula in Lemma 1. If *δx*_1 _is significantly smaller than  for almost all  ∈  having a non-negligible probability *P *(), Theorem 1 may be applied. For a general stochastic differential equation, it is hard to exactly indicate how much smaller *δx*_1 _must be compared to . This highly depends on the structure of the system and also on the accuracy being required for the solution. Nevertheless, as a rule of thumb, the theorem gives good approximations if  < 0.2. It is not necessary that this is true for every  ∈ , but the calculated density of the amplitude may differ from the real one for the amplitudes *χ *∈  for which the corresponding *δx*_1 _is not small enough. Nevertheless the accuracy of the amplitude distribution for other amplitudes is not affected (see proof).

• It may be possible to relax the assumption that the curvature of the level curves does not change its sign globally. Intuitively it seems to be sufficient for most systems that the curvature does not change its sign only locally around the states  ∈ , and that the amplitude in one oscillation period reaches its maximum in the area around . However this has to be shown for the system of interest explicitly.

• If the Lie derivative *L*_*f*_*P *of the density distribution *P *is not small enough, the net flux density  cannot be approximated to be tangential to the level curves of *P *anymore. Depending on the size of *L*_*f*_*P *this may lead to a significant bias in the calculation of the amplitude distribution for certain systems. In this case, the derivation of the amplitude distribution would be more involved, and also depend on the term , starting from an appropriate modification of (66) in the appendix.

### 2.4. The Damped Harmonic Oscillator in the Presence of Additive Gaussian White Noise

#### 2.4.1. Equations and Properties of the System

In this section we give a motivational example for the application of Theorem 1 by discussing the behavior of the damped harmonic oscillator in the presence of additive Gaussian white noise. The oscillator satisfies the following Langevin equation:(9a)

with Γ_*i*_, *i *∈ {1, 2} uncorrelated, statistical independent Gaussian white noise with zero mean and variance of one, *σ *∈ ^+ ^and *k *∈ .

It is easy to show that the deterministic system (*σ *= 0) is asymptotically stable for *k *> 0. The eigenvalues of the system matrix(10)

are at λ_1,2 _= -*k *± *i*, therefore the deterministic system will show damped oscillations upon perturbation from its steady state.

#### 2.4.2. Calculation of the Density Distribution

For *σ *> 0 we can calculate with the help of the multivariate Fokker-Planck equation the density distribution of *x*_*i*_, *i *∈ {1, 2}, in the state space. The general multivariate Fokker-Planck equation (see, for example, [3, p. 193 ff.] or [13, p. 96 ff.]) is given by(11)

with (**x**) and *B*_*i*, *j *_(**x**) *> *0 real functions describing the influence of the system dynamics and the noise on the distribution. The diffusion term **B **may be calculated to(12)

with **Σ **the matrix of the noise terms of (1). We call (11) linear if ∀_*i*_ (**x**) is a linear function in **x **and ∀*i*, *j B*_*i*, *j*_(**x**) is constant. Then (11) can be simplified to(13)

For the system (9), *A*_*i*, *j *_are given by the system matrix *A *from (10), and *B*_*i*, *j *_are the elements of the matrix **B **= *σ*^2^*I*_2_, where *I*_2 _is the second order identity matrix. To get the stationary density distribution, we set . Following [3], the stationary density distribution for the linear Fokker-Planck equation (13) is given by(14)

with ⟨ **x **⟩ the mean value of **x **and **Ξ **the matrix of the second moments of P, which is the solution of the equation **AΞ **+ Ξ**A**^*T *^+ **B **= 0. In system (9), ⟨ **x **⟩ = **0 **and Ξ may be calculated to . The state of the system is therefore Gaussian distributed with the maximum of the density being at the EP.

#### 2.4.3. Determination of the Amplitude Distribution

To get the amplitude distribution *P*_*A*_(*χ*) of the oscillations of the harmonic oscillator (9), we apply Theorem 1 after checking the assumptions made in the theorem. Notice that due to the easy structure of this first example and its symmetry, we are always measuring the oscillations in a direction tangential to a principal axis of the distribution. Therefore (*χ*) = (*χ*, 0)^*T *^for all *χ*. Because of this it is easy to calculate the necessary variables with the help of the definitions (8a)-(8e) to(15a)

After some calculations we obtain(16)

For *s *= 10 and *k *= 0.01, *δx*_1 _is getting small compared to  for  ≥ 20, so our approximation should at least hold for every amplitude greater or equal to 20. The results will nevertheless show that we even get good estimations for amplitudes much smaller than 20.

Due to the linearity of the oscillator (9) and the symmetry of the harmonic oscillator, we can determine the amplitude distribution by utilizing formula (5) as(17)

**Figure 4:**
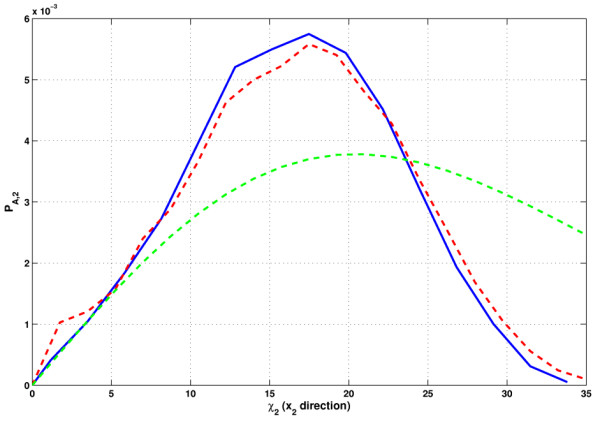
**Amplitude distribution of the oscillations of the damped harmonic oscillator**. Amplitude distribution of the oscillations of *x*_2 _of the damped harmonic oscillator (9) in the presence of white noise with autocorrelation *σ *= 10 and *k *= 0.01. The solid curve corresponds to the data from simulation, the dashed curve is the prediction according to Theorem 1. The simulation was run over a time scale of 100000 s which corresponds to around 16000 oscillation periods.

For *s *= 10 and *k *= 0.01 the predicted amplitude distribution *P*_*A *_is plotted over the amplitude *χ *in Figure 4. For comparison we also displayed the amplitude distribution of the stochastic oscillations as measured in a long term simulation run.

This easy example was discussed to give an insight into the reasons for stochastic oscillations. In the next section, we show the practical applicability of our algorithm by predicting the amplitude distribution of a complex biochemical system and therewith give an example of a more biologically relevant application for the results of the paper.

### 2.5. Oscillations in the MAP Kinase Signaling Cascade

**Figure 5:**
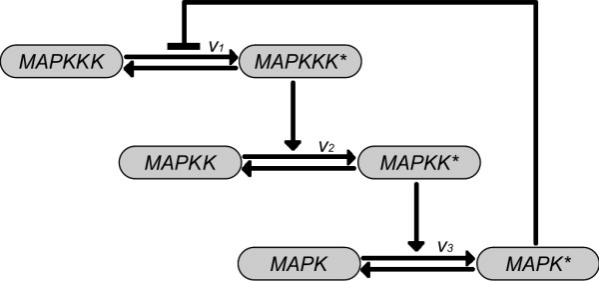
**Schematic view on the MAP kinase signaling cascade**. Schematic view on the MAP kinase signaling cascade. The species with the stars (*) are the phosphorylated versions of the species without stars. MAPKKK* catalyzes the phosphorylation of MAPKK, MAPKK* in turn catalyzes the phosphorylation of MAPK. A negative feedback is given by the repression of the activation of MAPKKK by MAPK*.

In the following section we apply the algorithm developed in this paper to a realistic example from biochemical signal transduction. A frequent module in many eucaryotic cells from yeast to mammals is the mitogen activated protein (MAP) kinase signaling cascade. MAPK cascades are typically activated by extracellular stimuli such as growth factors, and regulate the activity of various genes, thereby provoking a cellular response to the applied stimulus. Many important cellular functions such as differentiation, proliferation and death are controlled by MAPK cascades [14]. MAPK cascades consist of three layers of kinases, where each kinase phosphorylates and thereby activates the kinase on the next layer, as shown in Figure 5. The kinases are named MAPKKK, MAPKK, and MAPK, in the order of activation, and the phosphorylated, active forms are denoted with a star. The basic structure may be complemented by additional feedback interconnections, giving rise to deterministic limit cycle oscillations [15]. More recently, oscillations in the MAPK cascade have been determined experimentally in yeast cells, where the oscillatory activity of the MAPK controls periodic changes in cell shape during the mating process [16].

#### 2.5.1. The Deterministic Model

For this example, we use a basic ODE model of the MAP kinase signaling cascade. The model contains a negative feedback interconnection from the last kinase MAPK to the activation of the first kinase MAPKKK [15]. We use a simpler model compared to the one in [15], and therefore sustained oscillations do not occur in the deterministic version of the model considered here.

Denote *x*_1_, *x*_2 _and *x*_3 _the concentrations of MAPKKK*, MAPKK* and MAPK*, respectively. Using three conservation relations(18a)

we get the deterministic description of the system using mass balancing with Michaelis-Menten reaction kinetics:(19a)

with the reaction rates given by(20a)

The parameter sets for the phosphorylation (*k*_*ij*_) and for the dephosphorylation (*p*_*kl*_) are given in Table 1.

**Table 1:** Parameter Set for the MAP Kinase Signaling Cascade

Parameter	Value	Unit
*k*_11_	0.256	1/s
*k*_12_	0.0872	1/nM
*k*_13_	0.1144	1/nM
*k*_21_	0.4229·10^-2^	1/s
*k*_22_	0.0347	1/nM
*k*_31_	0.2049·10^-2^	1/s
*k*_32_	0.0243	1/nM
*p*_11_	0.1003	1/s
*p*_12_	0.1428	1/nM
*p*_21_	0.1218	1/s
*p*_22_	0.0794	1/nM
*p*_31_	0.1343	1/s
*p*_32_	0.0167	1/nM

The biochemically relevant equilibrium point **x**_0 _of the system is computed as(21a)

We can determine the local stability of this EP by analyzing the Jacobian *A *=  of the system's dynamics evaluated at the EP. The eigenvalues of *A *are obtained as(22a)

and thus the deterministic system is locally asymptotically stable.

#### 2.5.2. The Stochastic Model

**Figure 6:**
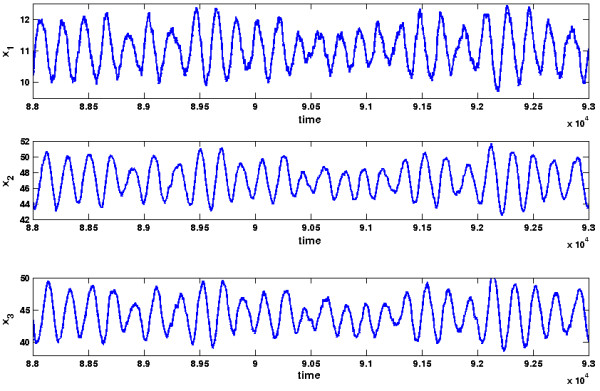
**Oscillations of the MAP kinase signaling cascade**. Plot of the oscillations of the MAP kinase signaling cascade. The plot is the result of a stochastic simulation via the Direct Gillespie approach with Dizzy.

For validation purposes and to get a first insight into the system's dynamics, we did stochastic simulations of the biochemical network model of the MAPK cascade as described by (19). We used the stochastic simulation software Dizzy by the Institute for Systems Biology [17], which is able to do stochastic simulations of models not only with mass action kinetics, but also with arbitrary kinetics such as Michaelis-Menten kinetics, which are used in our model. For the simulations we assumed a cell volume of *V *= 1 pl, which corresponds to the absolute number of molecules of each enzyme of(23a)

where *N*_*A *_is the Avogadro constant. We provide a typical plot of the oscillations of *x*_1_, *x*_2 _and *x*_3 _against the time in Figure 6. As can be seen from the plot, the oscillations don't vanish as the deterministic calculations suggested, but are sustained oscillations with a determined amplitude range. In the following we will show that these oscillations can be analyzed with the help of Theorem 1.

As discussed in [11] it is possible, under certain assumptions, to describe a system of biochemical reactions by a set of stochastic differential equations, giving rise to the so called Langevin approximation. These assumptions are, roughly speaking, fulfilled if the amount of entities of each species does not get too small, like in our system. The Langevin approximation of the MAPK cascade model (Figure 5) is given by(24a)

with Γ_*i*, *f *_and Γ_*j*, *b*_, *i*, *j *∈ {1, 2, 3} uncorrelated, statistical independent Gaussian white noise with zero mean and a variance of one, *N*_*A *_the Avogadro constant and *V *the cell volume. This system corresponds to the deterministic one with an additional noise part for every reaction added, with a standard deviation corresponding to the square root of the magnitude of the reaction rate divided by the cell volume. The factors of the white noises,  and  with *i*, *j *∈ {1, 2, 3}, are monotone in **x **and don't change significantly in an area around the EP, so that we can approximate them with their steady state values. Afterwards we can combine each function's noise terms by adding their variances , leading to the following equations:(25a)

with ,  and  for a cell volume of *V *= 1 pl.

#### 2.5.3. Transformation and Model Reduction

The theoretical results in this paper have been developped for two-dimensional systems only. In higher order systems stochastic oscillations may appear on a two dimensional manifold in the state space. Such a system can be reduced to an order two system by computing the slow manifold, making use of a time scale separation [18]. Following this approach, we first transform the system (25) to(26a)

with *z*_1 _∈  the coordinate of the fast and **z_slow _**= (*z*_2_, *z*_3_)^*T*^ the coordinates of the slow manifold, ψ_*fast *_and ^ψ^_**slow **_= (ψ_*slow*, 1_, *ψ*_*slow*, 2_)^*T *^vectors of polynomials of order two and higher, *ϕ*_**fast **_and *ϕ*_**slow **_matrices of the noise strength and Γ = (Γ_1_, *Γ*_2_, *Γ*_3_)^*T *^the noise vector of system (25).

The transformation needs to be done in such a way that the absolute values of the real parts of the eigenvalues of *A*_*fast *_are much larger than these of **A**_**slow**_. To this end, we compute the eigenvectors corresponding to the eigenvalues (22a)-(22b) as(27a)

The two eigenvectors with non-zero imaginary parts, *e*_2 _and *e*_3_, are both eigenvectors corresponding to eigenvalues having real parts with small absolute values. Therefore it is straightforward to define the desired transformation as(28)

with the transformation matrix **T **= (**e**_1_, **e**_2 _+ **e**_3_, (**e**_2 _- **e**_3_)·*i*). We can get the reaction rates of the transformed system by calculating(29)

Because all of the eigenvalues (22a)-(22b) have non-zero real parts, the Hartman-Grobman theorem states that there exists a local transformation **z **= **H**(*ζ*) such that the system in *ζ*-coordinates obeys the differential equation(30)

Because in the *ζ*-system there exists a slow manifold spanned by the eigenvectors corresponding to the two eigenvalues with real parts having low absolute values, in the **z**-system there exists a slow manifold, too (see [19]). We now search the function *z*_1 _= *m*(*z*_2_, *z*_3_) which describes the dependence of the states on the manifold. We approximate this function with a truncated Taylor series expansion:(31)

**Figure 7:**
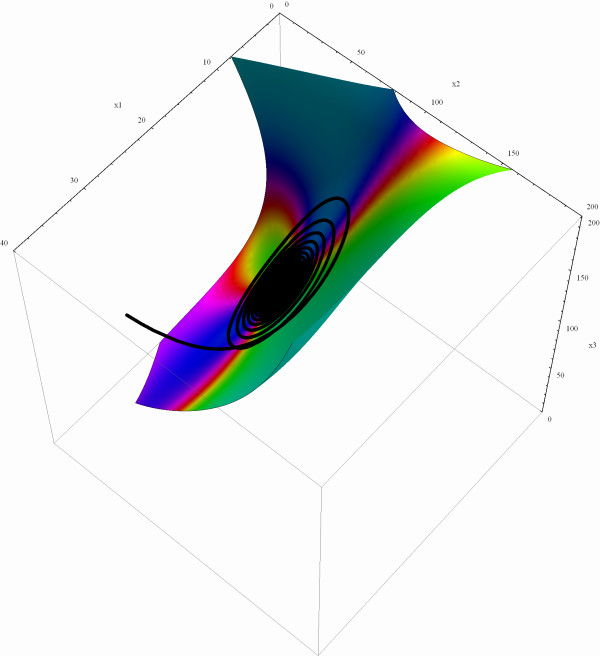
**Slow Manifold of the MAP kinase signaling cascade**. Slow manifold of (20) calculated up to the order of four. The black curve is a representative trajectory of the deterministic system.

We substitute (31) in  = *v*_*z*,1 _and can therewith calculate the coefficients *a*_*j*, *k *_(see Table 2). The resulting manifold in the original **x**-coordinates is shown in Figure 7. The figure also includes an example trajectory. As can be seen, this trajectory first converges exponentially fast to an ϵ-neighborhood of the slow manifold and afterwards moves on it towards the EP.

**Table 2:** Parameter Set of the Slow Manifold of the MAP Kinase Signaling Cascade

Parameter	Value	Parameter	Value	Parameter	Value
*a*_0,0_	0	*a*_0,2_	0.01800	*a*_4,0_	7.327·10^-8^
*a*_1,0_	0	*a*_3,0_	-8.476·10^-6^	*a*_3,1_	-8.191·10^-7^
*a*_0,1_	0	*a*_2,1_	3.625·10^-5^	*a*_2,2_	-8.162·10^-7^
*a*_2,0_	0.003604	*a*_1,2_	2.425·10^-4^	*a*_1,3_	3.951·10^-6^
*a*_1,1_	0.01102	*a*_0,3_	5.750·10^-4^	*a*_0,4_	1.785·10^-5^

The slow manifold is attractive enough so that realizations of the stochastic system (25) will stay close to it. Due to this, it is sufficient to just take the oscillations on the slow manifold into account and therewith simplify the problem to two dimensions. We substitute the formula of the slow manifold (31) in the differential equations for *z*_2 _and *z*_3 _(29) and get a two dimensional reduced description of the system(32a)

to which Theorem 1 can be applied.

#### 2.5.4. Calculation of the Density Distribution

As a first approach to obtain the stationary density distribution *P *(**x**), we make use of a linear approximation of the reduced system (32) around the EP. Taking this approach allows us to evaluate how well our method works with such an approximation, where the stationary density distribution can be obtained with minimal computational effort. For other systems, or if a high precision of the result is required, it could however also be necessary to solve the nonlinear Fokker-Planck equations numerically.

The linear approximation of the reduced system (32) is given by(33)

with the system matrix(34)

and **Γ **the vector of the disturbances Γ_*i*_, *i *∈ {1, 2, 3}, of the original system (25).

From the coordinate transformation *T *applied to the original system, **Σ**_η _is determined by(35)

where diag(*σ*_*i*_) is a diagonal matrix with the diagonal elements *σ*_*i *_being the standard deviations of the stochastic terms in the system (25). For the system (33) with *V *= 1 pl, **Σ**_η _is obtained as(36)

**Figure 8:**
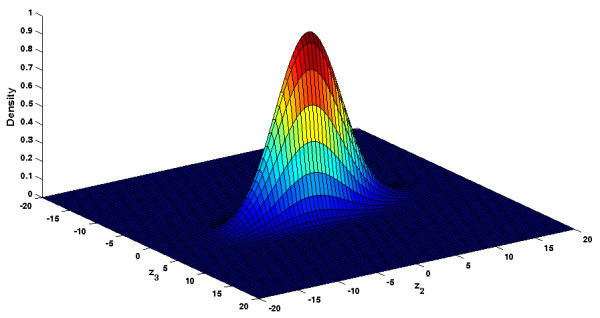
**Linear distribution of the state of the MAP kinase signaling cascade**. Linear density distribution of the MAP kinase signaling cascade on the slow manifold as calculated in (37). Because we linearized the system, the distribution is Gaussian.

Following the same approach as in the previous example, the stationary density distribution is obtained as(37)

with(38)

for a cell with a volume of *V *= 1 pl (see Figure 8).

#### 2.5.5. Determination of the Amplitude Distribution

With the preliminary work of the preceding sections we are now able to calculate the amplitude distribution according to Theorem 1. We consider the states **x**_χ _= (*χ*, *α*, *β*)^*T *^with *a*, *β *∈  not yet specified. These states are transformed into the *z*-coordinates by(39)

The probability of the amplitude *χ *can now be calculated with the formula(40)

under the constraint that **z**_*χ *_has to lie on the slow manifold. This corresponds to that **z**_*χ *_has to satisfy (31), which gives us a dependency of *β *on *α*. This dependency can be obtained numerically by solving a convex optimization problem.

**Figure 9:**
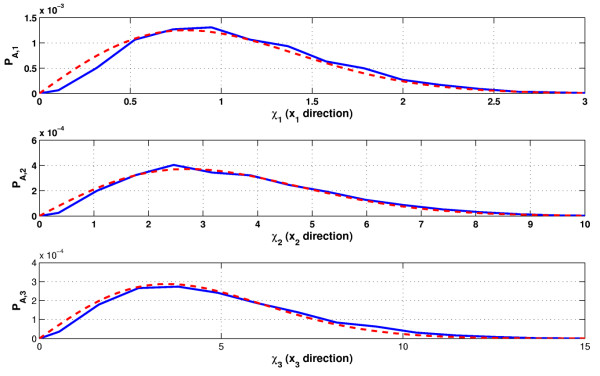
**Linear amplitude distribution of the oscillations of the MAP kinase signaling cascade**. Linear amplitude distribution of the oscillations of the MAP kinase signaling cascade. The solid curve corresponds to data determined by stochastic simulation of the nonlinear system (24), the dashed curve is the prediction according to the calculations in this section.

The resulting amplitude distributions in each of the three original coordinate directions *x*_*i*_, *i *∈ {1, 2, 3} are shown in Figure 9. For comparison, the figure also contains measured amplitude distributions, which are the results of a long term simulation via the Direct Gillespie approach with Dizzy with a simulation over 900000 steps.

As can be seen in Figure 9, the simulation results fit the calculated predictions very well. However, it seems that we tend to underestimate the amplitude of the oscillations by a small amount. This can be explained as a result of Lemma 1, which states that the amplitude of a realization going through the state  lies with a high probability in [,  + *δx*_1_], whereas we estimate its value by the lower end  of this interval (see appendix). The tendency to underestimate the amplitude seen in Figure 9 seems to be a direct result of neglecting the value of *δx*_1_. Further discussion of this point can be found in the conclusions.

We also want to mention that we compare the results obtained from our method to stochastic Gillespie simulations, and not to realizations of the Langevin equation (24). The reason herefor is that Gillespie simulations better predict the behavior of biochemical networks and are thus the method of choice. However in the derivation of the amplitude distribution we approximated the stochasticity of the system using white noise terms. The results of both methods to describe the intrinsic noise of the system may under certain circumstances lead to different results, which may be a further explanation of the small bias between the measured and the theoretical predicted amplitude distribution in Figure 9. Furthermore some bias may be explained due to the linearization of the system around its steady state.

#### 2.5.6. Numerical Approach

Sometimes it is not adequate anymore to analyze the linearized system and one has to analyze the original nonlinear one. Although there might be several special cases where the probability distribution *P *of a system of nonlinear stochastic differential equations is analytically computable, this is not possible in the general case. As a consequence there is only the possibility to obtain the solutions numerically. To give an example how to solve a problem with the numerical approach, we decided to analyze the same system as in the preceding section, as defined in (24), except of changing the cell volume to *V *= 0.017 pl, which corresponds to  of the original cell volume. Because the variance of the disturbances is highly dependent on the amount of entities of the different protein species, the shape of the solution changes dramatically in a way that we cannot get good results anymore by analyzing the linearized system.

**Figure 10:**
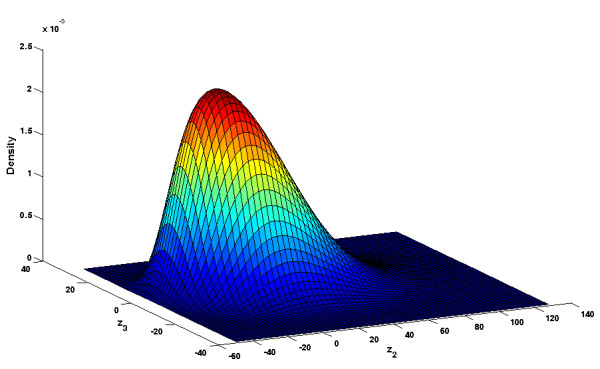
**Nonlinear Distribution of the state of the MAP kinase signaling cascade**. Density distribution of the MAP kinase cascade on the slow manifold (31).

To calculate the density distribution of the nonlinear system we have to solve the nonlinear Fokker-Planck equation (11). We therefore applied the algorithm developed in [20]. The shape of the density distribution, which is non-Gaussian, is shown in Figure 10.

**Figure 11:**
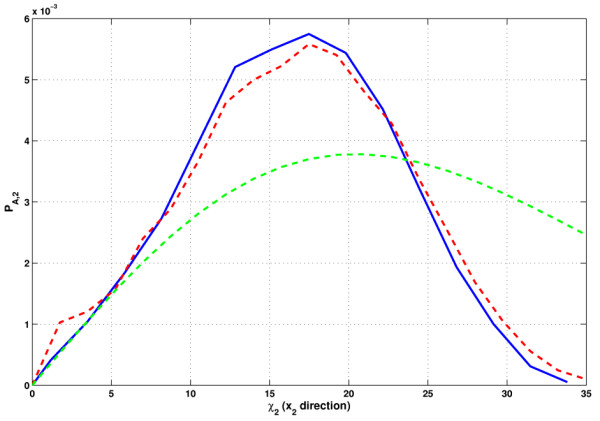
**Nonlinear amplitude distribution of the oscillations of the MAP kinase signaling cascade**. Nonlinear amplitude distribution of the oscillations of the MAP kinase signaling cascade in the negative *x*_2_-direction. The solid curve corresponds to data experimentally measured, the dashed red curve is the prediction according to the calculations in this section and the dash-dotted green curve is the outcome we get by calculating the amplitude distribution of the linearized system.

From the density distribution *P *(**x**), we can compute the amplitude distribution *P*_*A*_(*χ*) with the method proposed in this paper. In this example, we analyze the amplitude distribution *P*_*A*_(*χ*) of *x*_2 _for peak amplitudes below the equilibrium point values, i.e.  = (0, -1, 0)^T^, because then the effect of the nonlinearities is even higher than in the other direction. The reason for this is quite simple: The amount of entities of each species is not allowed to become negative. As can be seen in Figure 11 the amplitude distribution, as obtained by applying Theorem 1, quite well fits the predicted results gained from a long time simulation while the predicted values of the linearized system are far away from these values.

## 3. Conclusion

We introduce a method to determine the amplitude distribution of a wide class of linear and nonlinear stochastic systems given by (1), which display sustained stochastic oscillations. The method is applicable to systems where a stationary density distributions exists and can be computed either analytically or numerically.

The method is based on computing the flux density of realizations for the states where the tangent on the level curve of the density distribution is normal to the direction in which the oscillations are measured. We showed that under certain conditions this flux density is directly proportional to the probability of an oscillation with an adequate amplitude to occur. Our results can be used in the analysis of systems being influenced by strong internal or external noise, as we often find them in biophysical problems.

As already discussed at the end of the second example, for certain systems the calculated amplitude distribution may contain a small bias depending on the exact structure of the system and on how well the the assumption necessary for the application of our method are satisfied. For the wide class of nonlinear systems analyzed we could obtain as a result that realizations being at state  will most likely have an amplitude lying in [,  + *δx*_1_] as stated in Lemma 1. By requiring *δx*_1 _to be small we justified to approximate the amplitude by . However, if for specific systems the distribution in [,  + *δx*_1_] can be calculated or estimated, it would be possible to reduce the bias for systems where *δx*_1 _is small but not negligible. Such an extension would lead to a more refined approximation of the amplitude distribution.

Stochastic oscillations may occur in biological systems not only as a disturbing side effect, but also in a constructive manner [21,22]. This is not because stochastic oscillations have great benefits over more "traditional" types of oscillations like deterministic limit cycles, but due to the fact that there seems to be no reason for a preference of deterministic limit cycles. In biophysical systems, the type of oscillations we study in this paper often occurs at parameter values in the vicinity of a Hopf bifurcation in the deterministic part of the model [23]. This is important in the field of robustness analysis of biological networks [24], because stochastic oscillations can possibly improve the robustness of oscillations in a network. In this respect, our method to compute the oscillation amplitude may be helpful in order to characterize a parameter region in which either deterministic or stochastic oscillations of a comparable amplitude occur robustly.

## Appendix

### Proof for Lemma 1

In this section we give a short proof for Lemma 1. We therefore consider the level curve defined by  of the density distribution going through the state  ∈ .

In the following we argue that there exists a *δx*_1 _such that for a high probability an arbitrarily chosen realization of (1) being at state  at time *t *won't reach a state **x **with *x*_1 _>  + *δx*_1 _until the next oscillation and therefore the measured amplitude will lie in [,  + *δx*_1_] with *δx*_1 _positive and small but yet not further determined (see Figure 2). We first calculate the Taylor series expansion for *P *up to the order of two:(41)

After substituting the definitions made in the equations (8) and utilizing the knowledge that  vanishes because of the definition of , setting

, we get(42)

If we stay on a level curve, it must hold that *δP *= 0 and *δx*_1 _has to be a function of *δx*_2 _locally around . This function is approximated with another Taylor series expansion up to order two as(43)

with *k*_1_, *k*_2 _yet unknown constants. By substituting (43) in (42) we get(44)

Because the coefficients of *δx*_2 _and  must vanish independently for (44) to hold, it follows that *k*_1 _= 0 and *k*_2 _= , giving(45)

This means that we may approximate a level curve of the density distribution locally by a parabola. For small deviations and small times *t *it is possible to make some approximations for (1). First we may approximate **Σ **(**x**) in an area around  by the constant(46)

We can rewrite the norm of the first row elements of  by(47)

which can be easily validated by the definitions of *L *and *a *given in the equations (8). We furthermore approximate the second element of **f**(**x**) in an area around  by(48)

Putting everything together we get a one dimensional Wiener process for the movement of (1) in the direction of *x*_2 _for small times Δ*t*:(49)

with **Δx**(*t*) = **x**(*t*) - . We approximate the average displacement in the direction of *x*_1 _by combination of the average displacement of this Wiener process (⟨ *x*_2_(*t*)⟩ = *νt*) with equation (45):(50)

Because the stochastic part of (49) may be neglected (see Assumption 4), we can get a linear one dimensional stochastic equation for the movement of (1) in the direction of *x*_1 _for small times *t*:(51)

It can be shown [10, p. 129 ff.] that the variance of (51) evolves as(52)

We may think of the solution of (51) as a growing Gaussian distribution moving along the trajectory of the deterministic system (see Figure 2). We now determine the maximal value of Δ*x*_1_(*t*) that a realization of (1) starting at  may have if it evolves in an area around the average displacement determined by the standard deviation . The maximal value of Δ*x*_1_(*t*) is time dependent and grows in the beginning due to the stochastic part of (51), but afterwards shrinks due to the deterministic part. It reaches its maximal value when the time derivatives of the mean value and of the standard deviation have the same absolute value:(53)

We can now calculate *δx*_1 _to(55)

For a Gaussian distribution it is true that 68.3% of all realizations stay in an area around the average displacement determined by the standard deviation. This means that only  of the realizations reach an amplitude greater than  + *δx*_1 _after passing . Because it is also possible for a realization to have its maximal value in the direction of *x*_1 _before passing , the overall probability for a realization to have an amplitude lower than  + *δx*_1 _is(57)

In the same way it can be shown that 95.5% of all realizations have an amplitude lower than  + .

### Proof for Lemma 2

Assume a small unit cell with the edge lengths *dr *and *dφ *with its center being **x **and the level curve of *P *through **x **given and going through the center of the edges with the lengths *dr *(see Figure 3). If *dr *and *dφ *are small, we may assume the density distribution *P *and the values **f**(**x**) and **Σ**(**x**) of (1) as constant inside the unit cell. Due to the small size of the Lie-derivative of *P *along *f*, the movement normal to the level curve is dominated by the stochastic part of (1). The net fluxes *ϕ*_1 _and *ϕ*_3 _trough the edges 1 and 3 are vanishing and therefore the overall flux is tangential to the level curve. The net flux *ϕ*_2 _(*ϕ*_4_), trough the edges 2 (the edge 4) can be determined by integration (see, for example, [[25], p. 622]):(58)

By letting *dr *and *dφ *go to zero the absolute value of the net flux density ||(**x**)|| follows:(61)

### Proof for Theorem 1

Because the density distribution *P *is smooth and the curvature of its level curves does not change its sign, there exists exactly one state  ∈  with  = *χ *for every *χ *∈ (0, ∞).  is a smooth simple curve in the state space which can be parameterized by(62)

with  an unknown but smooth function in *χ*. We can get a term proportional to the average number of oscillations of all realizations per time unit  if we determine the total net flux of realizations *ϕ*_*t *_through this curve:(63)

with  the normalized normal vector on  in . We may determine the probability *P*_*A*_(*χ*) of the amplitude *χ *∈ (0, ∞) by multiplying the flux density of realizations  with Ω (, *χ*), the probability that a oscillation of a realization contributing to the flux density at  has the amplitude, and integrating over all  ∈ :(64)

From the condition  we get(65)

Because of Lemma 2 we know that the flux density  is directed along the *x*_2_-axis and has the absolute value *P *() *ν*(). Therefore we can calculate (64) to(66)

Although we do not know the exact shape of Ω (, *χ*), which may be complicated to calculate, we know a lower and an upper bound for Ω. The lower bound is given due to the fact that Ω must be zero for *χ *<, because if (1) goes through the state , it has at least the amplitude of this state. An upper bound for almost all realizations is given by Lemma 1 with  + . We may therefore conclude that Ω is approximately zero for *χ *being outside of the interval [,  + ], which simplifies (67) to(68)

If *δx*_1 _is small, we can assume *P *and *ν *being approximately constant in the integration interval, which simplifies (68) to(69)

For small *δx*_1 _the integral of Ω over  is approximately 1. The result of this last approximation is given by Theorem 1.
